# Mission Bambini and the “Children's Heart Program”: The Challenge of Congenital Heart Disease Among Emergency and Education

**DOI:** 10.3389/fped.2021.705607

**Published:** 2022-02-03

**Authors:** Stefano M. Marianeschi, Giulia Albano, Nicola Viola, Alberto Barenghi, Alex Gusella

**Affiliations:** ^1^Niguarda Ca' Granda Hospital, Milan, Italy; ^2^Mission Bambini Organizzazione Non Governativa, Milan, Italy; ^3^Congenital Cardiac Services, University Hospital Southampton National Health Service Foundation Trust, Southampton, United Kingdom

**Keywords:** congenital heart disease, cardiac surgery, cardiology, NGOs, multidisciplinary education, healthcare staff training models, volunteering

## Abstract

Congenital heart disease is defined as abnormality in the cardiovascular structure or function that is present at birth and it is the most common cause of congenital anomalies. Approximately 90% of more than 1,000,000 children born per year with congenital heart disease worldwide receive suboptimal care or have no access to care at all. Furthermore, the mortality is likely underreported in Low-and Middle-Income Countries. Mission Bambini Foundation is an Italian NGO founded in 2000, aiming at “helping and supporting children who are poor, sick, without education or physically and morally abused” in Italy and worldwide. In 20 years, through 1.700 projects, 1.4 million children have been supported in 75 Countries. In 2005, Mission Bambini launched the “Children's Heart Program,” based on long-term partnerships and on medical/surgical volunteering, in order to provide multidisciplinary education and training and technical support.

## Introduction

Congenital heart disease represents a major global health problem.

The estimate of 8 per 1,000 live births is generally accepted as the best approximation (1). **Every year**, out of the 130 million babies born in the world approximately, **more than 1 million are born with congenital heart disease**. It is interesting to note that studies of populations from low, lower-middle, and higher-middle income countries (based on the World Bank classification) seem to observe, except for some exceptions, a lower incidence of congenital heart disease than the accepted value if 8 per 1,000 (2). While in High-Income Countries congenital heart disease is easily diagnosed and successfully treated through cardiac surgery if necessary, Low-and Middle-Income Countries are often lacking trained medical staff and adequate health facilities. Furthermore, access to diagnosis is more difficult (3). Cardiological screenings and early diagnosis is decisive also for rheumatic heart disease.

The “Children's Heart Program” is one of the most important and representative programs of Mission Bambini, with the final goal of treating children with congenital heart disease at a local base in Low-and Middle-Income Countries, by working with local governments and local healthcare staff. The surgical treatment of congenital heart disease implies substantial resources, complex infrastructures and equipment and highly skilled professionals. Therefore, the involvement of the governmental institutions is crucial, in order to guarantee the sustainability of the service.

The **strategy** of the “Children's Heart Program” is based on the following three pillars:

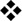
 **Life-saving cardiac surgery**: children suffering from heart disease receive life-saving cardiac treatment.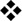 **Capacity building and training**: local partner hospitals are self-sufficient in treating heart diseases in children (diagnosis, surgical treatment and follow up).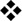 **Prevention**: regarding Rheumatic Heart Disease (RHD), we developed a program in Eritrea and Zambia to avoid the onset or worsening of RHD in children within 10 years of age. **Timely care** must be given to treat otorino-laryngeal infections. For this reason, children between 6 and 10 years undergo to **medical examinations and cardiological screening in schools** and/or health points.

The **main actions** realised to put into effect the strategy are referred to:

1) Carrying out **regular paediatric cardiac surgery camps in local hospitals (two camps per year per 5 years in each Country)** with highly skilled volunteer teams to perform paediatric cardiac surgery, with the goal to see and operate the largest possible number of children, particularly the most complex and urgent cases.

2) Defining and implementing a program of **theoretical and practical training**–during surgery or with specific training sessions–addressed to the medical and nursing local staff, aiming at transferring competencies and skills.

During our medical camps in 8 Countries, were trained 16 surgeons, 17 cardiologists and 20 anaesthesiologists. They were able to perform 1.354 interventions in 12 years.

3) Identifying and taking charge of **structural and instrumental needs of the local hospitals** in order to develop paediatric cardiac surgery on site.

In 15 years, through the “Children's Heart Program” it has been possible to reach children with heart defects in 19 Countries worldwide. Currently, the program is implemented in **9 Countries**: Albania, Eritrea, Italy, Nepal, Myanmar, Romania, Uganda, Zambia, and Zimbabwe.

Since the beginning, more than 2,300 children have been treated and saved, the 60% of which by the local medical teams. More than 20,000 underwent cardiological screening, of which almost 13,000 for Rheumatic Heart Disease prevention (4).

The most common congenital heart defect treated within the “Children's Heart Program” are Tetralogy of Fallot and ventricular and atrial septal defects, of medium complexity in terms of congenital heart surgery. For instance, the project carried out in Cambodia from 2012 to 2019 counts 53 Ventricular Septal Defects (VSD), 31 Tetralogy of Fallot (ToF), 21 Atrial Septal Defects (ASD) out of a total of 114 patients who underwent heart surgery (the 92% of the total treated cases).

The Program overall surgical mortality is 2%: 1,354 patients were operated by local teams with mortality of 3.1%, whilst 976 patients were operated during medical camps with mortality of 0.9%.

Regarding morbidity, we collected few data because the surgeries performed were of medium/simple complexity and life-saving (ASD, VSD, ToF) and the rate of re-operation was very low (0.2%). Moreover, most patients live in rural areas, so that the morbility was difficult to calculate because some of them were lost in the follow up.

With regard to hospitalisation, patients normally stay at the hospital an average of 5 days, of which 3 days in intensive care unit.

## Policy Options and Implications

The policy underlying the “Children's Heart Program” procedures and protocols for guiding decisions and achieving outcomes has changed over the years, both in terms of senior management decision making and operational aspects.

The experience gained has allowed Mission Bambini to modify and improve the strategy to increase the impact of the Program itself and to make it even more sustainable.

Mission Bambini implemented two different policies.

According to the first one, from 2005 to 2010, Mission Bambini acted as “simple” donor, supporting different autonomous volunteer medical teams and Italian no profit organisations, whose main aim was to surgically treat children with congenital heart disease on-site or in Italy.

The process to access the Mission Bambini donations implied the receiving of a formal application, discussed and evaluated by the Technical Committee of the Foundation (composed by internal and external members) on the basis of qualitative and quantitative criteria. If approved, the annual budget of the new project was defined and divided in various instalments, to be transferred according to the Ethical Contract stipulated between Mission Bambini and the partner, after receiving the periodic narrative and financial report.

The partnership was on multiyear basis, to be renewed annually, in order to ensure continuity to the project activities.

In the first 5 years of the “Children's Heart Program,” Mission Bambini supported 6 projects and contributed to treat 235 children born with heart defects, investing about 1,400 euros per patient.

In 2010, Mission Bambini started changing the policy of the Program, by introducing two new approaches:

a) The identification of local hospitals in Low-and-Middle Income Countries with a minimum of technical skills and knowledge on paediatric cardiac surgery as new partners to be financed.

In these cases, children with heart diseases could be diagnosed and treated in their own country, however surgery might be too expensive, making it unsustainable for families with a low or medium income or the medical facilities might not be adequate.

In these Countries, Mission Bambini covered the surgery costs for children of the neediest families and helped to improve and equip medical facilities, where necessary.

This type of intervention could also include the coverage of the costs for the follow up.

b) The implementation of its own medical camps, involving a group of volunteer healthcare professionals.

The long term aim of the partnership with local hospitals was for them to reach the autonomy in treating heart diseases in children, raising the level, and quality of the whole local sanitary system. During medical camps, the selected local healthcare staff worked alongside with the MB volunteer doctors, nurses, and technicians for the transfer of skills and know-how. To ensure continuity of the training activities, the medical camps were carried out twice a year.

Patients were eligible for heart surgery on the basis of urgency and difficulty level in performing the case. Apart for surgery, during the camp, among 20 and 40 children are screened, diagnosed or seen for follow up.

Normally, the first screening is conducted by the local hospital. Then the first day of the medical camp the two medical teams discuss the cases and finalise together the patients list for surgery.

The patients surgical final list reflects the balance between clinical needs of the patients and clinical skills of the local medical team. Moreover, in order to ensure successful surgeries, the patients selection must consider other elements, such as the efficiency of the intensive care unit (including the neonatal ITU), the availability of an ECMO program and the presence of a cardiologist and intensivist, to guarantee the well-being of patients once the medical camp has been completed.

Once these clinical needs and skills are balanced, it is necessary to match them also with the training/education aims.

Every medical professional worked with his/her local homologous colleague, in a continuous training *on the job*. Moreover, lectures and talks were given by the medical volunteers to the local staff, according to a program set before with the local hospital.

Thus, Mission Bambini switched **from being a donor Foundation to be an implementer Foundation**, taking charge of the ideation and execution of its own Program on congenital heart disease.

Nowadays, to include a Country/Hospital in the long-term collaboration to develop the paediatric cardiac surgery service, Mission Bambini organises an institutional and clinical assessment. If positive, a Memorandum of Understanding (MoU) is signed between Mission Bambini and the local hospital, under the endorsement of the local Ministry of Health.

The MoU states the mutual obligations and the common aims in terms of capacity building, surgeries to be performed, mortality rate, etc.

Mission Bambini ensures the compliance of the MoU and the monitoring and evaluation phases of the project.

All the logistics (travel, room and board, visa, travel insurance) is organised and funded by Mission Bambini. Mission Bambini ensures also the purchase of consumables, if needed and in order to guarantee the correct execution of the surgical interventions.

Currently, as implementer and funder of the “Children's Heart” Program, Mission Bambini:

- Develops long-term partnership with public local hospitals, involving the Public Institutions (Ministry of Health, Italian Embassy, local Universities…);
- Verifies the feasibility of paediatric cardiac surgery camps in the Countries;
- Reports outcomes and results to the stakeholders;
- Ensures monitoring and evaluation phases;
- Realises fundraising and communication activities;
- Plans paediatric cardiac surgery camps and organises logistics.

It was a gradual transition from the first to the second policy during the years, coming to involve 9 local hospitals.

From 2014, the “implementing” policy was enriched with new elements, due to the Mission Bambini's aim of enhancing the training and education for the local healthcare professionals.

In fact, while in North America and Europe there is approximately one congenital heart surgeon for three and one-half million individuals, in South America the ratio is of 1:6,500,000, in Asia of 1:25,000,000, and similarly in Africa of 1:38,000,000. This clearly demonstrates a large need for human resources (2).

Thus, Mission Bambini decided to support an annual scholarship worth 10,000 euros for a local healthcare professional selected among the partner hospitals, to complete the ≪Cardiac surgery, cardio anaesthesia, and cardiology≫ Master, at Milano Bicocca University, in collaboration with the International Heart School. Since then, six professionals (heart surgeons, cardiac anaesthesiologists, and cardiologists) attended the post-graduate course and came back to work at the hospital in their country.

Aware of the importance of training and education, in 2017 Mission Bambini has also designed a new training and education project within “Children's Heart” Program, aiming at enhancing the theoretical and technical competences of local professionals, in collaboration with the local Universities of Medicine, in order to fully train specialists who will perform at the highest level in their own specialty and will lead the progression of the local team and institutions.

In 15 years of the “Children's Heart” Program, Mission Bambini collected numerous lessons learned and decided to elaborate a Theory of Change, to better understand how to keep improving its policy, identifying the ultimate change to be reached as the reduction of the mortality rate in developing countries of children affected by congenital or rheumatic heart diseases, with particular attention to the less privileged population.

The “Children's Heart” Program is today a replicable model, which contributes to raise the level of the paediatric cardiovascular surgery service and the quality of the whole local health system.

**Figure 1:**
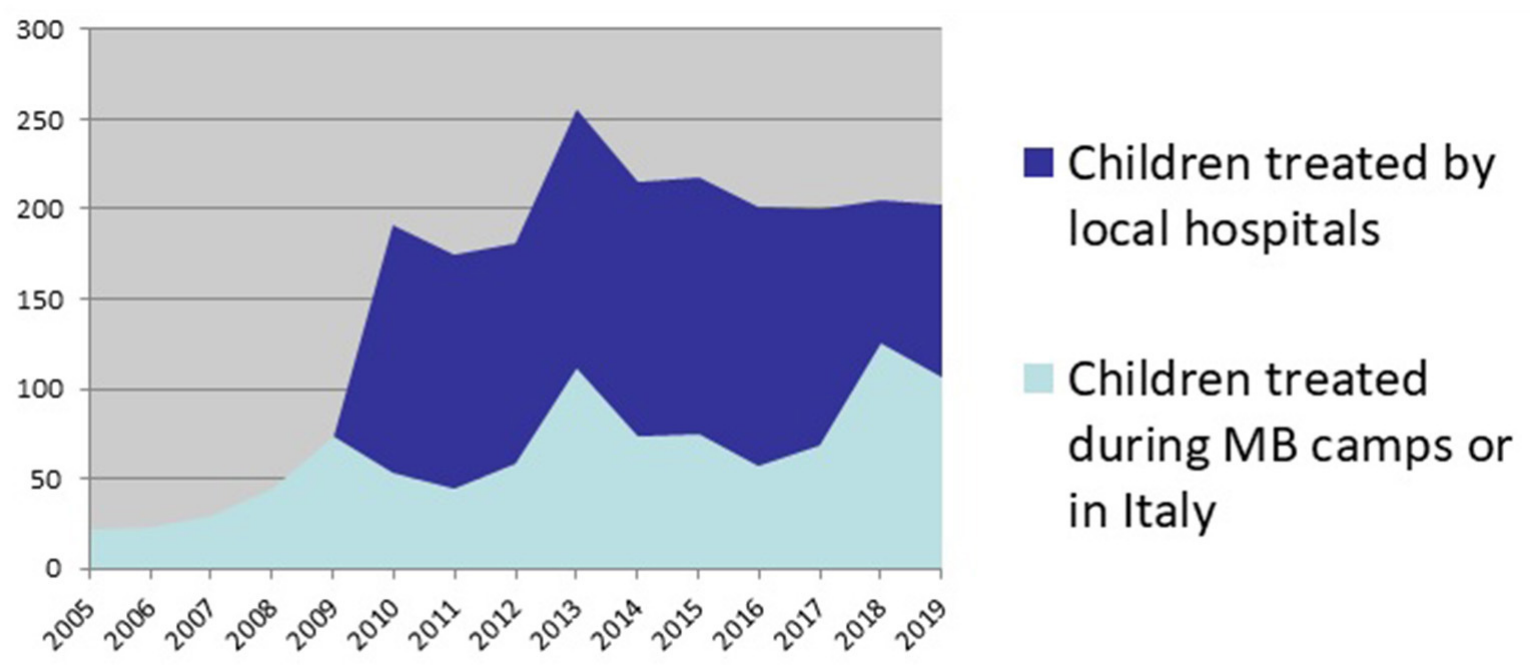
Children treated autonomously by local hospitals or by the MB volunteer healthcare staff.

The **implications** of the evolution from the first policy to the second one are numerous:

- Investing in the capacity building of local hospitals guarantees the sustainability of the paediatric cardiovascular surgery service and allows to reach and treat more children, as shown in the Figure 1.
- Treating children on-site by the local hospital is cost-effective for Mission Bambini. In fact, a paediatric cardiac surgery performed during a medical camp costs to Mission Bambini about 1,500 euros, instead of 800 euros if Mission Bambini finances the same cardiac surgery performed by the local team.
- The training and education play a key role in developing a paediatric cardiovascular surgery service. During the years, Mission Bambini delivered 6,051 h of training on the job and 1,428 h of lectures, involving about 500 local health professionals.
- The clinic and training activities are possible thanks to the generosity of highly skilled health professionals in paediatric cardiac surgery who volunteers for Mission Bambini, from Italy, Spain, UK, USA. Every volunteer represents a valuable resource for Mission Bambini, shares its values and vision and operates in accordance with them to support the “Children's Heart” Program aims and objectives.

During the years, Mission Bambini faced limitations in terms of medical resources and skilled personnel on site. Moreover, it is very challenging to realise a complete screening and to follow the patient through the entire post-operative course in a short period of time like a normal medical camp.

It is also essential to consider the possible threats related to the development and implementation of the Program.

Changes of government and political and economic instability of Countries are among the most likely risks to be faced. Moreover, local professionals might look for a better work conditions after being trained and leave the hospital partner.

In order to mitigate the mentioned risks, an effective collaboration and engagement must be achieved at all levels, together with a careful shared planning.

The Mission Bambini experience demonstrates that the more accurate assessment and monitoring and evaluation effort are, the more a paediatric cardiac surgery program can be developed with favourable results.

## Actionable Recommendations

What makes the “Children's Heart” Program a successful program are elements that can also be shared and replicated by other NGOs and no profit organisations.

The first recommendation is taking the lead of the process and be accountable to the stakeholders.

For instance, Mission Bambini guarantees a multiple-year implementation for all the projects in the different Countries, the monitoring and evaluation phases of the various projects, the sharing of the Program outputs and outcomes, the formal authorizations to carry out the paediatric cardiac surgery camps as planned, the planning and logistics of the medical camps scheduled for the year, the safety of the team when abroad, the visa and travel insurance procedures, the coverage of the costs for all the team members related to flights, accommodation and stay of the medical team, visa, travel insurance, and the costs for medical equipment, if needed.

The second recommendation is about creating and maintaining valued networks.

Mission Bambini currently collaborates with 6 local hospitals in Africa, Asia and Europe and with 7 local no profit organisations for Rheumatic Heart Disease prevention, access to adequate healthcare and follow-up.

Mission Bambini can count on more than 100 volunteer healthcare professionals from Italy, UK, Spain, and USA.

As a volunteer of Mission Bambini, every medical professional becomes part of a network of international professionals who donate their time and competences in the field of paediatric cardiac surgery. This means that everyone could experience professional growth, thanks to the opportunity to work with colleagues from different backgrounds.

Every volunteer shares its professional experience to train, theoretically and practically, local partners' medical staff and at the same time he/she experiences cultural exchange, working side by side with local medical staff in a different context in terms of hospital facilities, work practises and job procedures.

Involving public institutions, such as Ministry of Health, Embassies, etc. is also crucial.

The third recommendation is to take into great consideration the key role of education and training for the sustainability of the projects.

For instance, thanks to the scholarships supported by Mission Bambini, at the local hospital partner in Uganda is currently working a multidisciplinary team, with a shared academic background.

## Conclusions

The evolution of the “Children's Heart” Program and its policy is likely to continue in the next future.

Mission Bambini will continue to ensure the monitoring and the scaling up of the Program, to keep learning from the experience and to keep developing new strategies.

Many things can be certainly improved, but at the same time the conditions due to the Covid-19 pandemic are demonstrating that the “Children's Heart” Program's approach is effective.

In fact, since the travels abroad are still not allowed, Mission Bambini cannot organise the medical camps. However, thanks to the job done in the past years in terms of education and training, in 2020 the local hospitals performed paediatric cardiac surgeries autonomously and Mission Bambini planned new activities and created new tools, such as an online platform to keep ensuring the training remotely.

## Author Contributions

All authors listed have made a substantial, direct, and intellectual contribution to the work and approved it for publication.

## Conflict of Interest

The authors declare that the research was conducted in the absence of any commercial or financial relationships that could be construed as a potential conflict of interest.

## Publisher's Note

All claims expressed in this article are solely those of the authors and do not necessarily represent those of their affiliated organizations, or those of the publisher, the editors and the reviewers. Any product that may be evaluated in this article, or claim that may be made by its manufacturer, is not guaranteed or endorsed by the publisher.
